# A modified neo-vagina procedure in a low resource urogynecological unit: a case report of a 21 year old with Mayer-Rokitansky-Küster-Hauser (mrkh) Syndrome operated at Mbarara referral hospital, Southwestern Uganda

**DOI:** 10.1186/s12894-017-0258-7

**Published:** 2017-08-29

**Authors:** Musa Kayondo, Joseph Njagi, Peter Kivuniike Mukasa, Tom Margolis

**Affiliations:** 1 Faculty of Medicine, Mbarara University of Science and Technology, P.O. BOX 1410 Mbarara, Uganda; 2 Department of Obstetrics and Gynecology, Mbarara Regional Referral Hospital, P.O. BOX 40 Mbarara, Uganda; 3 United Nation Fund for Population Activities, Kampala, Uganda; 4 Department of Obstetrics and Gynecology, University of California, Los Angeles, USA; 5 Medled Medical Missions, Burlingame, California, USA

**Keywords:** Vaginal agenesis, Mayer-Rokitansky-Küster-Hauser syndrome, Skin grafts, Neovagina, Vaginal molds, Case reports

## Abstract

**Background:**

Although vaginal agenesis as may occur in Mayer-Rokitansky-Küster-Hauser (MRKH) syndrome is a rare condition, it is associated with not only anatomical problems but also serious psychological and social problems like painful sexual intercourse, primary amenorrhea and infertility. Surgery, which is aimed at reconstruction of a vagina of adequate length and width to serve the function, is the main method of treatment. Many methods for vaginal reconstruction have been described but each has its complications and limitations. The most commonly preferred procedure for treating this condition is the McIndoe vaginoplasty which involves dissection into the recto-vesical space, inserting two split thickness skin grafts folded over a mold in this newly created space and regular dilatation of the neovagina postoperatively to avoid stenosis. However surgeons with this expertise in this part of the world are rare to find and where they are available, the special molds on which to fold the skin grafts into the neovaginal space are not readily available.

**Case presentation:**

A 21-year-old female with vaginal agenesis was operated on using a modification of the McIndoe procedure using a cylinder of a 60cm^3^ syringe as a vaginal mold/form and kept in place. We left a Foley in place for 10 days and we did a dye test after removing the syringe to ensure that there was no leakage resulting from fistula formation.

**Conclusion:**

The operation was successful and on subsequent monthly reviews of the patient, she has a patent functional vagina of about 9 cm in length at 8 months after the operation with resumption of sexual intercourse.

## Background

The vagina is an important organ in females not only forming a part of the birth canal but also serving as the main organ for sexual intercourse. Congenital absence of the vagina which usually occurs in Mayer-Rokitansky-Küster-Hauser syndrome, Androgen insensitivity syndrome and other embryological disorders usually presents with social problems ranging from failure to have sexual penetration, painful coitus and sometimes broken marriages which if not taken care of often lead to serious psychological problems like depression and rejection [1]. Such women require construction of a new vagina (neo-vaginal procedure) so that they can at least have sexual pleasure [1–3]. In most centers these women are turned away due to lack of surgeons with expertise to handle this problem. Even in places where the expertise is available, resources required for this complex procedure are not available. Known conventional neo-vaginal surgical procedures include McIndoe vaginoplasty, rectosigmoid vaginoplasty and the modified vacchietti [1, 3, 4]. The most widely used among these methods is the classical McIndoe which involves dissection and creation of a neovagina in the rectovesical space and insertion of 2 skin grafts from the inguinal regions folded over a vaginal mold (form) into this space. Split-thickness skin grafts have over the time been used in this operation. The skin is harvested from a region of the body, which is hair free notably from the inguinal region, buttocks and inner thighs. The skin is harvested using an air powered or electrical dermatome. The harvested skin is folded over a foam mold and inserted in the newly dissected recto-vesical space. Closure of the donor site is done with a vicryl 2/0 suture inserted subcutaneously to ensure hemostasis and cosmesis. The vaginal mold prevents restenosis of the neovagina and is kept in for a period of at least 10 days [2–5]. These procedures are relatively complex and inappropriate in a low resource setting like our hospital because the vaginal molds to prevent restenosis of the neovagina are not readily available and neither are the electric or air powered dermatomes for harvesting skin.

Another procedure that has been described for treatment of MRKH syndrome is the Vacchietti procedure. This procedure utilizes a laparoscope to produce a neovagina whose dimensions are comparable to those of a normal vagina. In this procedure, a small sphere that is made of plastic (“olive”) is fixed on the vaginal dimple; the strings on the olive are passed through the mucosa of the vagina into the peritoneal cavity and through the navel. The strings are attached on to a traction device, which is then adjusted daily such that the “olive” is pulled inwards. This serial pull stretches the vagina by approximately 1 cm per day hence forming a vagina, which is approximately 7 cm wide, and 7 cm in depth. With regards to restoration of vaginal anatomy and function in patients with MRKH syndrome, the laparoscopic Vacchietti technique has better success rates when compared to other treatments [6–8]. Laparoscopy is not available in this part of the world hence this procedure is not possible at our hospital.

Therefore in this case we designed a relatively easier procedure (modified McIndoe) where we harvested 2 full thickness skin flaps from the inguinal region using a scalpel instead of an electric dermatome which was folded on to a cylinder of a 60cm^3^ syringe to act as the vaginal mold to prevent restenosis of the reconstructed vagina in a 21-year-old woman with vaginal agenesis due to Rokitansky’s syndrome. A surgeon, with skill in vaginal surgery can easily do this procedure.

## Case presentation

**Fig. 1:**
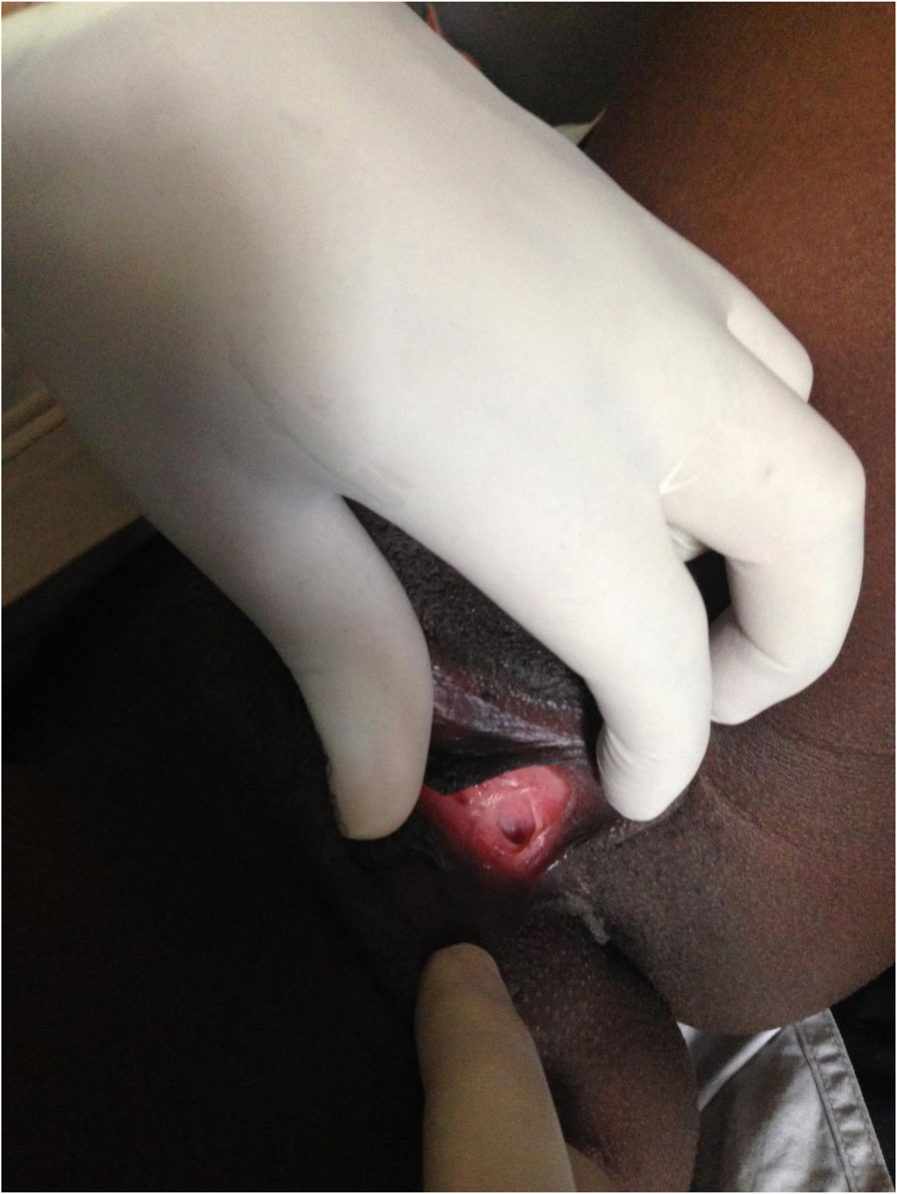
Showing the Vaginal dimple of the 21 year old patient with vaginal agenesis before surgery

A 21-year-old woman was admitted to the gynecology ward of Mbarara Regional Referral Hospital (MRRH) with primary amenorrhea, failure to conceive and painful coitus. All these had affected her marriage of three years to a point of divorce. On evaluation she was found to have a normal female genotype with well-developed secondary sexual characteristics. Pelvic examination revealed a vaginal dimple with no palpable uterus (Fig. 1). An abdominal ultrasound scan revealed presence of both ovaries with a streak like or rudimentary uterus. The rest of the pelvic organs were normal. A preoperative diagnosis of vaginal agenesis due to MRKH syndrome was made. A decision to do surgery of creating a new vagina by using a modification of the McIndoe procedure was decided. Counseling of the patient about the surgical procedure with emphasis on the expectations was done. We explained to the patient that the procedure would improve her sexual life but would have no effect on her ability to conceive which she accepted and consented to the operation. Bowel preparation using soap enema plus overnight fasting was done prior to the operation. After induction of anesthesia, the patient was put in lithotomy position. The vulva was cleaned with antiseptic solution then draped. A urethral catheter of size 16 was inserted into the bladder. A transverse incision was made on the vaginal dimple to open into the rectovesical space. Bunt dissection was continued until a length of about 9 cm and 3 cm in width was achieved. A methylene blue dye was introduced into the bladder to make sure that there was no accidental bladder injury and a finger was also introduced into the rectum to ensure that there was no rectal injury.

Two full thickness hair free folds of skin measuring 12 by 6 cm in length and width were harvested from each lateral side of the abdominal wall starting from the anterior superior iliac spines. We used the lateral side instead of the skin over the inguinal ligament as done in the McIndoe method because our patient was too skinny for us to get adequate length and width of skin in this area. The folds were prepared by first immersing them in normal saline then the subcutaneous fat from each of the folds was removed using a sharp scissor. The assistant closed the sites from which the grafts were harvested using nylon 2/0 sutures interrupted vertical matrix as the lead surgeon prepared the grafts.

**Fig. 2:**
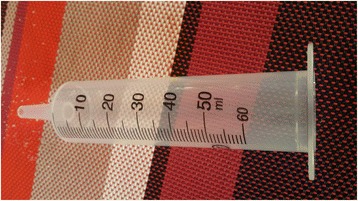
The cylinder of a 60cm^3^ syringe, which was used as a mold instead of the conventional vaginal form on which the skin grafts were folded to prevent stenosis of the neovagina

Unlike in the McIndoe method were the skin flaps are folded on a vaginal mold/form, here we used the cylinder of a 60 ml bladder syringe to act as a stent for the grafts in place of the vaginal form due to lack of the foam rubber to make a vaginal form (Fig. 2). The tip of the syringe was cut off before it was inserted. The skin grafts were folded on the cylinder of the syringe with the epidermal sides lying on the cylinder using interrupted vicryl 3/0 sutures. The excess lengths of the grafts were trimmed off.

**Fig. 3:**
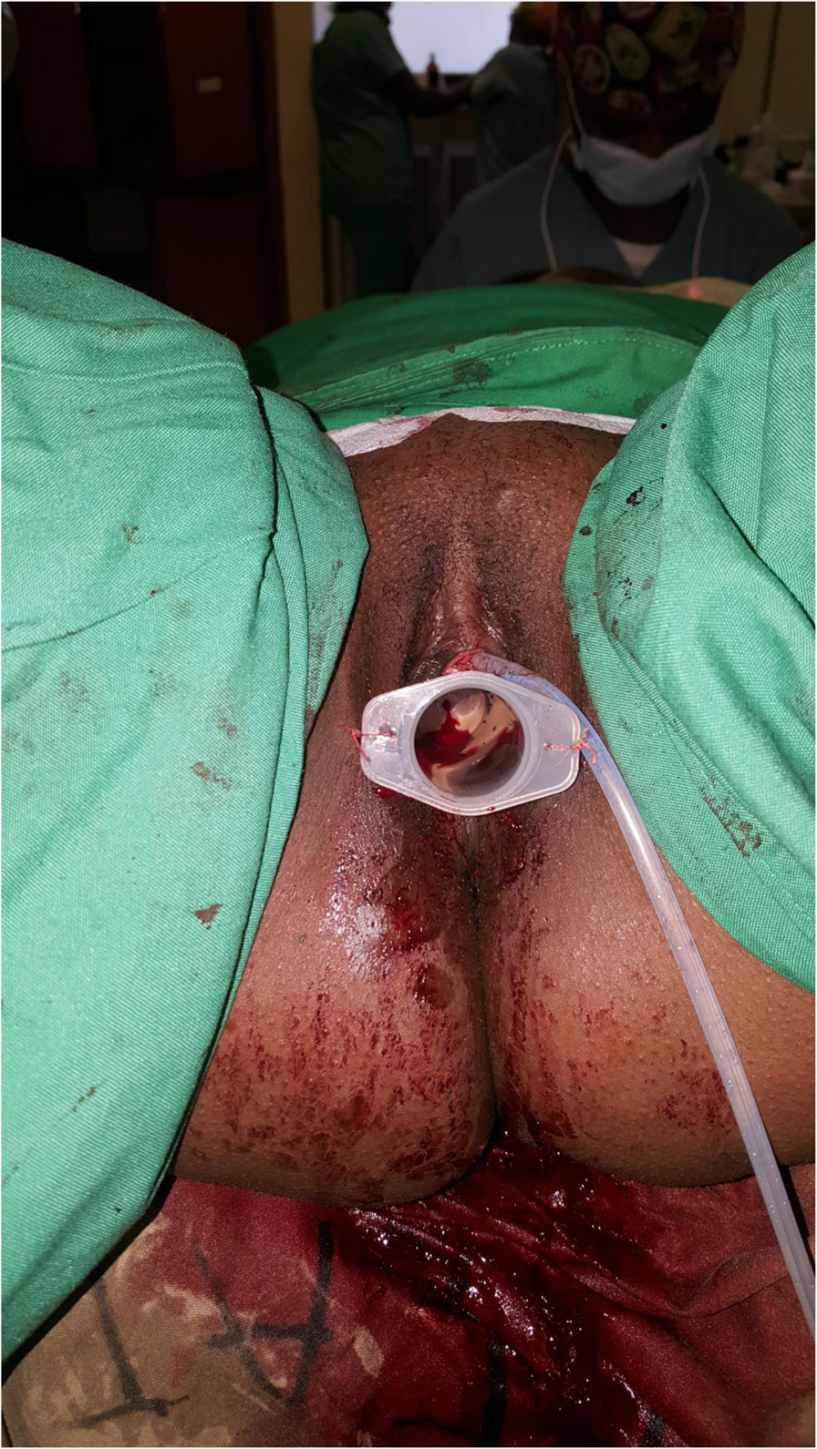
Showing the cylinder of the syringe inserted into the neovagina and fixed on to the labia majora by a stitch on either side for 10 days to prevent restenosis

The graft with its cylinder was then carefully inserted into the newly created rectovesical space after achieving hemostasis. In order to secure the graft in place, the cylinder of the syringe was fixed to the labia majora on either side using a nylon 2/0 suture unlike in the McIndoe method where the labia are sutured in the midline. The cylinder stent was maintained for 10 days to keep the neo-vagina patent. The urethral catheter was also kept in for the same duration to ease the patient’s voiding and to also rest the bladder and urethra to guard against pressure necrosis that could be caused by the prolonged pressure on the urethra by the cylinder hence causing a fistula. A low residue diet was prescribed for the patient to prevent constipation and hard stools. She was also put on oral antibiotics and analgesia to prevent post surgical infection and pain for about 5 days. We used antibiotics, which were readily available in the hospital (Ceftriaxone and metronidazole). The labial stitches anchoring the cylinder stent were removed on the 10th postoperative day on the ward. She was examined and there was no any postoperative complication like infection, graft rejection or bleeding (Fig. 3). A methylene blue test was done before the Foley catheter was removed to ensure that there was no fistula formed as a result of pressure necrosis by the cylinder on the urethra. After removal of the cylinder stent she was taught self-serial dilatation using a condom rolled on to a candle to prevent stricture of the neovagina. She was advised to do this self-dilatation for at least 3 months.

**Fig. 4:**
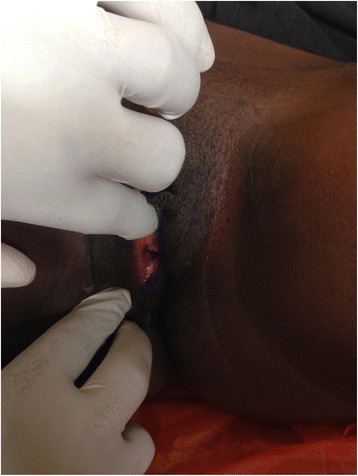
Opening to the newly reconstructed vagina on the 11th post operative day after removal of the cylinder that acted like the vaginal stent/mold

**Fig. 5:**
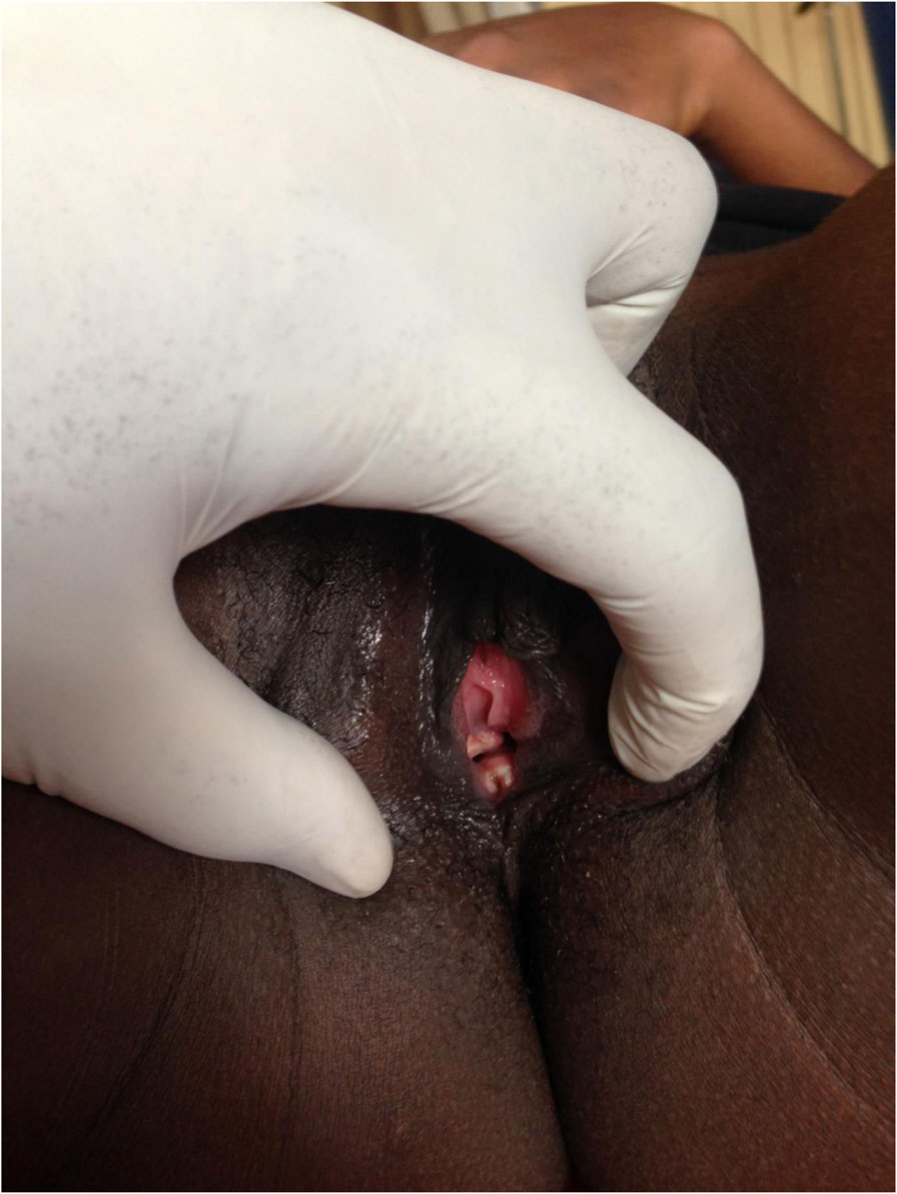
Appearance of the Neovagina at 3 months post operative

**Fig. 6:**
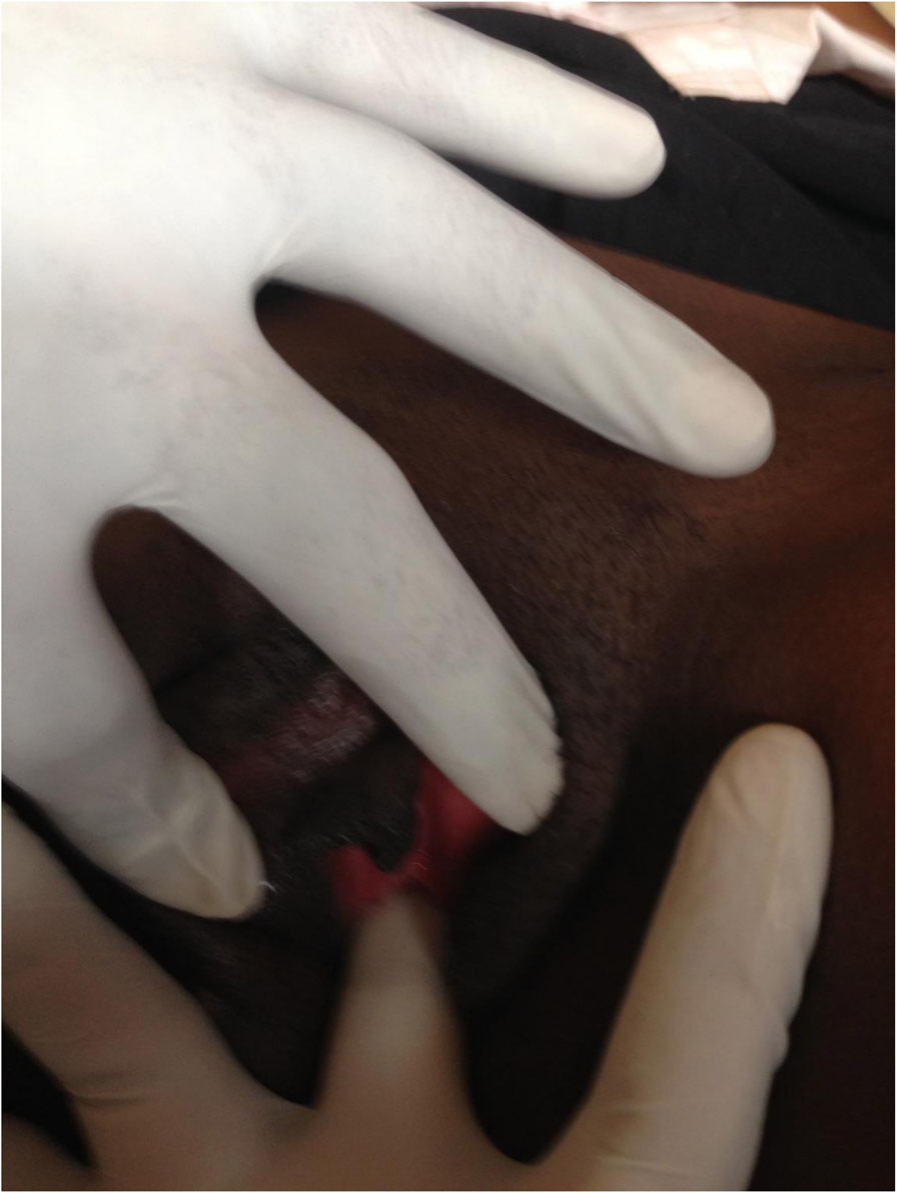
The neovagina still patent at 3 months post operation and able to admit at least one finger

She was discharged with a patent neovagina and the skin donor sites had healed after removal of the skin sutures. She has been followed up every 2 months for the last 8 months after operation and on examination the neovagina is patent (Figs. 4, 5 and 6) and she has resumed sexual intercourse, which has really improved her marital life.

## Discussion

Although vaginal dysgenesis seems to be a rare condition with reported incidences ranging from 1 in 4000 to 1 in 10,000 females, it is associated with adverse psychological and social effects in women [1]. Therefore the surgery to rectify this problem not only does it restore the anatomy but it also improves the psychological and social state of these girls and women like enabling them to enjoy penetrative sex thus preventing divorce [1, 3, 9].

In low resource countries like Uganda such women find it hard to get any medical help because there are a few or no surgeons with the expertise to handle these conditions. Even where expertise is available, the equipment and supplies like vaginal molds (forms) are not easy to come by.

In this case of the 21 year old with vaginal dysgenesis we started off the procedure same way as in the conventional McIndoe method. The difference of our procedure from the classical McIndoe is that we used full thickness skin flaps instead of the split skin grafts. The full thickness grafts are easier to harvest than the split thickness ones when an electrical dermatome is not readily available like in our case. The full thickness grafts have also been found to take a longer time to shrink during the postoperative period when compared to the split thickness grafts. This therefore shortens the time required in utilization of the vaginal foam [3–5]. Therefore in our case we kept the stent in situ for only 10 days because we used the full thickness skin grafts yet it would have stayed in longer if we had used the split thickness skin grafts which is usually uncomfortable to the patient.

In contrast to the McIndoe, we introduced methylene blue dye in the bladder to make sure that there was no bladder perforation during dissection of the rectovesical space. We also harvested skin folds from the hairless skin area above the anterior superior iliac spine in contrast to the McIndoe method, which uses the skin over the inguinal region. This was because our patient was very skinny that we couldn’t get enough skin over the inguinal region. It proved to be enough skin and a good area for harvesting skin in these circumstances.

The McIndoe method uses a specifically designed vaginal mold from foam rubber to prevent restenosis of the reconstructed vagina [2, 4, 5]. Since we couldn’t get the special vaginal mold, we improvised by using a cylinder of a 60cm^3^ syringe. This is the one that acted as a mold for the skin grafts inserted into the neovagina. These syringes are very easy to come by in our hospital; they cause minimal discomfort to the patient and can easily be removed after 10 days without distorting the integrity of the skin grafts in the neovagina.

In the surgical procedure we have described, we did not have to suture the labia in the midline to prevent the vaginal mold from falling out as is done in the McIndoe method. This reduced the postoperative pain usually encountered in McIndoe because of the sutured labia hence our patient was able to ambulate early and recovery was faster. We also removed the vaginal stent after 10 days compared to the 12 to 14 days mentioned in the McIndoe method hence reducing on the period of hospital stay of the patient.

Our modified procedure is less superior when compared to the Vacchietti laparoscopic technique in regard to anatomical and functional outcome of the newly created vagina [7, 8]. But in this part of the world, laparoscopy is not readily available making the Vacchietti procedure inappropriate for a hospital in this type of setting. This makes our modified procedure ideal for our hospital in a low resource setting.

## Conclusion

In patients with vaginal agenesis in low resource settings, a modification of the McIndoe method where a cylinder of a 60cm^3^ syringe acts as a stent/vaginal mold for 10 days without suturing the labia to keep it in place can be done successfully with little chance of vaginal restenosis in the future.

The postoperative pain and length of stay in hospital associated with this method is less when compared to the conventional McIndoe procedure.
